# The ΙgΕ-epitome viewed through the eyes of human monoclonal ΙgΕ

**DOI:** 10.1186/2045-7022-4-S2-P28

**Published:** 2014-03-17

**Authors:** Mattias Levin, Mats Ohlin

**Affiliations:** 1 Lund University, Department of Immunotechnology, Lund, Sweden

## Background

Despite the key role of IgE as the allergen recognizer at the initiation of allergic responses our knowledge on the molecular interactions between human IgE and allergen is limited. To date, most studies aiming at identification of IgE-reactive epitopes have had to rely on polyclonal IgE-preparations, non-human antibodies or antibodies of isotypes other than IgE. However, due to their intrinsic ability to recognize highly relevant, in the context of allergy, epitopes on allergens, allergen-specific human monoclonal IgE, represented by recombinant human antibody fragments, offer an opportunity to decipher the IgE-epitome of major allergens with precision and at high resolution. Such information may guide us in development of novel therapeutic candidates and better diagnostic tools.

## Methods

We have isolated a large pool of antibody variable domain fragments, specific for several major allergens, from the IgE repertoires of allergic donors. Three sets of such binders, targeting the major birch pollen allergen Bet v 1 (n = 4) and the major timothy grass pollen allergens Phl p 1 (n = 5) and Phl p 5 (n = 10), and their interactions with respective allergen have been studied by immunochemical methodologies.

## Results

We have, using our own technology and through collaborations, been able to define multiple non-overlapping IgE-reactive epitopes on all three investigated allergens. Two novel epitopes on Bet v 1 were identified, which allowed for identification of single amino acid residues with crucial importance for the interaction between human IgE and allergens in the PR-10 protein family. Further, it was shown that the relatively small allergen Phl p 5 carries a complex composition of IgE-reactive epitopes, providing an explanation to its high allergenicity. The less complex epitope composition on Phl p 1, on the other hand, with an IgE-binding hot-spot located on the surface of its immunodominant C-terminal domain, allowed us to, in a rational manner, design a hypoallergenic group 1 grass pollen allergen fragment, with the potential to be used as a safer alternative to the today used extracts in specific immunotherapy.

## Conclusion

We here summarize data that show the great usefulness of human monoclonal IgE as a tool in studies of the molecular interactions taking place at the initiation of allergic responses, and how information gained from such studies can aid us in, among other things, the design of novel hypoallergenic allergen variants.

